# Study of two MTA cements 

**DOI:** 10.4317/medoral.19936

**Published:** 2014-09-30

**Authors:** Esther Berástegui, Eduard Valmaseda-Castellón, Vicente Faus, María-Luisa Ballester, Leonardo Berini-Aytés

**Affiliations:** 1 DMD, PhD, Professor. University of Barcelona .Department of Endodontics. School of Dentistry. Barcelona, Researcher of the IDIBELL Institute. Barcelona; 2 DDS, PhD, Professor. University of Barcelona. Department of Endodontics. School of Dentistry. Barcelona, Researcher of the IDIBELL Institute. Barcelona; 3 DMD, PhD, Professor. University of Valencia; 4 Assistant Professor. University of Barcelona. Researcher of the IDIBELL Institute. Barcelona; 5 DMD, PhD, Professor. University of Barcelona. Professor of the Master Degree on Oral Surgery and Orofacial implantology. University of Barcelona. Researcher of the IDIBELL Institute. Barcelona

## Abstract

Introduction: To determine and compare the pH, conductivity and calcium release of an experimental Portland cement (PE) consisting of trioxid mineral aggregate and a comercially available modified Portland cement (C.P.M.) after 1, 2, 3, 4, 8, 10, 15 and 30 days. Material and Methods: Cements were mixed following the manufacturer’s instructions, with a powder: liquid ratio of 3:1. Each cement was placed in 12 PVC tubes 1 mm in diameter and 10 mm in length and allowed to set. Four empty tubes were used as negative controls. Tubes were submerged in plastic flasks containing 10 ml deionized water and stored at 37ºC and 100% humidity. After 1, 2, 3, 4, 8, 10, 15 and 30 days tubes were removed from the flasks and these were refilled with deionized water. We measured pH, conductivity and calcium content of the recovered solution. Data were analyzed using repeated measures ANOVA. Results: pH was 0.3 units more alkaline with PE cement (p=0.023). pH experienced a slight decrease with time (p<0.001), independently of the cement type (p>0.05). Conductivity of PE and CPM cements diminished at 4 days and almost recovered at 30 days (p<0.001). PE cement had a higher conductivity (p<0.001). Calcium release diminished from the first day and recovered at 30 days (p<0.001) similarly for both cements (p>0.05). Conclusions: PE cement raised pH slightly more and had higher conductivity than CPM. Calcium release diminished after the first day and recovered at 30 days, similarly for both cements.

** Key words:**Mineral trioxide aggregate, pH, electrical conductivity, periapical surgery.

## Introduction

Lee *et al.* (1) in 1993 described for the first time a new dental cement for use in various clinical situations. The material was a composite or aggregate (conglomerate) or Mineral Trioxide Aggregate (MTA) of gray. After MTA was commercially available, other similar cements were launched. The behaviour in the biological environment or bio-compatibility and other desirable properties of the cements mentioned above and the new MTA-based cements, has been the object of on goingre search by many groups studying material properties and clinical endodontics.

Nowadays there are many reports on this material, although uncertainties still persist. In terms of the proportion of its components and the chemical nature and mechanism of their action, questions have also been raised, due to the complexity of its variations in the chemical composition, all of them apparently similar. Torabinejad *et al.* (2) described MTA as a fine gray powder of hydrophilic particles consisting of compounds tricalcium silicate, tricalcium oxide, tricalcium aluminate and silicate oxide. Several subsequent studies have shown that MTA is similar to ordinary Portland cement used in construction. Recently other cements have appeared on the market such as the new MTA Angelus (Soluções Dentistry, Londrina, Brazil) or the MTA called Modified Portland Cement or C.P.M. (Egeo S.R.L.MTM, Buenos Aires,Argentina). The manufacturer of this latter MTA has also commercialized an MTA based endodontic sealer or root canal cement (Endo-C.P.M.-Sealer). The mechanism of action of MTA is similar to that of calcium hydroxide (1). The antimicrobial activity is related to the release of hydroxyl ions, which increase the pH of the connective tissue and create an unfavorable environment for bacterial survival. On the other hand, the formation of a mineralized barrier is induced by the release of Ca 2+ ions into the surrounding tissue. MTA stimulates hard tissue formation and the starting mechanism seems to be the release of calcium ions into the tissues (3). These promising qualities of the MTA justify the need for further research on important parameters suchas changes in pH, conductivity and calcium ion release. The study objectives were to compare the pH, conductivity and the ability to release calcium ions in saline solution of a commercially available MTA cement, Modified Portland Cement or C.P.M. and a new experimental material based in the formulation of Portland cement (CEMEX CEM II / B-P-32,5N (UNE-EN-197-1.CE-0099; Sant Feliu de Llobregat, Spain) from 1 day to 30 days.

## Material and Methods

We compared a new experimental Portland (PE) cement (CEMEX CEM II / B-P-32,5N; UNE-EN-197-1.CE-0099, Sant Feliu de Llobregat, Barcelona) with Modified Portland Cement C.P.M.® (CPM) (Egeo S.R.L. MTM, Buenos Aires, Argentina). PE Cement consists of a powder of tricalcium and dicalcium silicate, tricalcium aluminate and tetracalciuma lumino ferrite. We prepared twenty-four cement samples (12 PE and 12CPM) by mixing 150 mg of cement with 50 ml of saline in a proportion 3:1, following the manufacturer’s instructions. We measured powder with a precision scale (Adventurer Ohaus; Ohaus Corp., Pine Brook, NJ, USA). We used a glass plate and aplastic spatula for mixing to avoid metal contamination. We placed each cement sample into a PVC tube 10 mm long and with a diameter of 1 mm.

We sealed PVC tubes and allowed them to set for 60 minutes at 37ºC and 100% relative humidity, before submerging samples in 10 ml of deionized water, stored in a glassflask. After 24 hours, we carefully removed the tubes and placed them into another flask with the same amount of deionized water. This procedure was again repeated after 2, 3, 4, 8, 10, 15 and 30 days for each sample. Thus, 8 saline samples for each tube were recovered, which rendered a total of 96 saline solution samples for each cement. Four flasks containing PVC tubes without cement were processed as negative controls. We stored the negative controls in saline and recovered the saline solution only at day 1 and 30, to rule out any change of chemical properties of the saline solution not attributable to the cement. We placed the recovered fluid in plastic flasks for pH and conductivity measurements. We measured conductivity and pH with the pH testing device Hanna hi 9811-5 (Eutech instruments, Santiago de Chile, Chile). We repeated each measurement 8 times and used the mean value of the 8 measurements. Conductivity was expressed in μS/cm. The detection of calcium ions followed a standard protocol. We shaked the flasks before measurement. We added 15 μl (a 1% of the total volume) of 69 % nitric acid (HNO3) to each tube using an automated pipette, in order to stabilize the samples. We stored the tubes in a refrigerator before analysis. We detected calcium ions using inductively coupled plasma optical emission spectrometry (ICP-OES) (Spectrometer Perkin-Elmer, Optima 3200-RL; Buenos Aires; Argentina). Values were also the mean of 8 measurements. The results were expressed in ppm. We used SPSS 15.0 for Windows for data analysis (SPSS Inc; Chicago; IL; USA). The statistical test was a repeated measures ANOVA. The within-subject variable was timeand the between-subject variable was the cement. We chose the Greenhouse-Geissercorrection of the degrees of freedom if the Mauchly test ruled out sphericity. Differences were considered significant if *p*<0.05.

## Results

**Figure 1:**
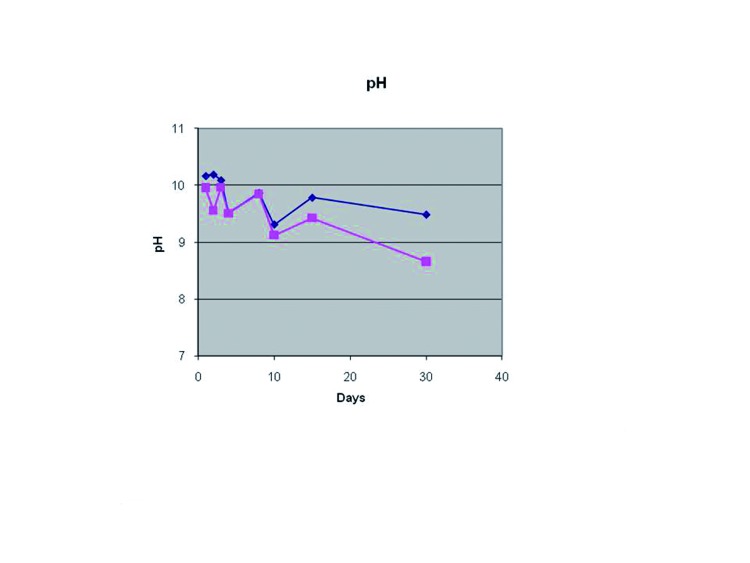
Comparison between PE and C.P.M. of obtained values of pH as a function of immersion time.

Fluid pH was markedly alcaline, with PE cement being 0.3 pH units (95 % CI of the difference: 0.04 to 0.54 pH units) more alkaline than CPM cement (F=5.953; df=1;=0.023). pH significantly changed across time (F=7.199; df=7; p=1.88•10-7) but this change was similar for both cements (F=1.157; df=7; *p*=0.331) (Fig. 1).

**Figure 2:**
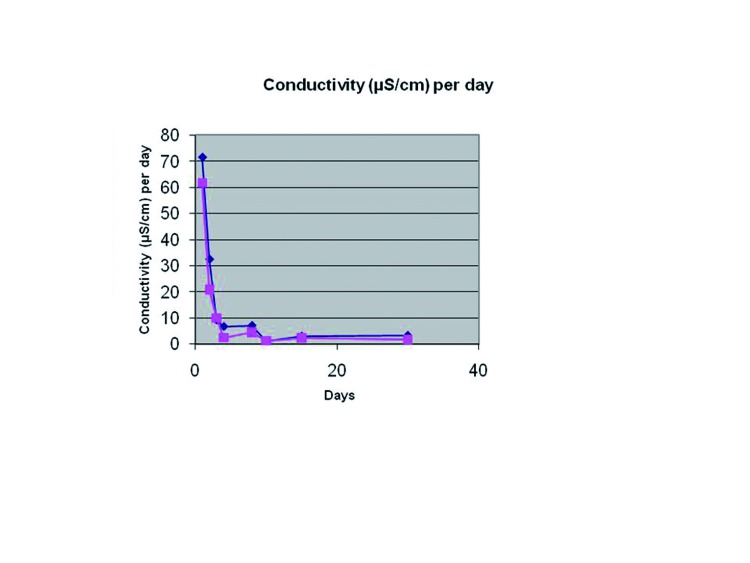
Comparison between PE and C.P.M. of obtained values of electrical conductivity as a function of immersion time.

Fluid conductivity was 7.60 μS/cm higher (95 % CI of the difference: 4.21 to 11.0 μS/cm) in PE cement samples (F=21.623; df=1; *p*=0.0001). Conductivity significantly changed across time (F=51.723; df=3.058; *p*=1.32•10-17), experiencing a marked reduction in the first days and then a slight increase at 1 month. This differences acrosstime were similar for both cements (F=1.665; df=3.058; *p*=0.182) (Fig. 2).

**Figure 3:**
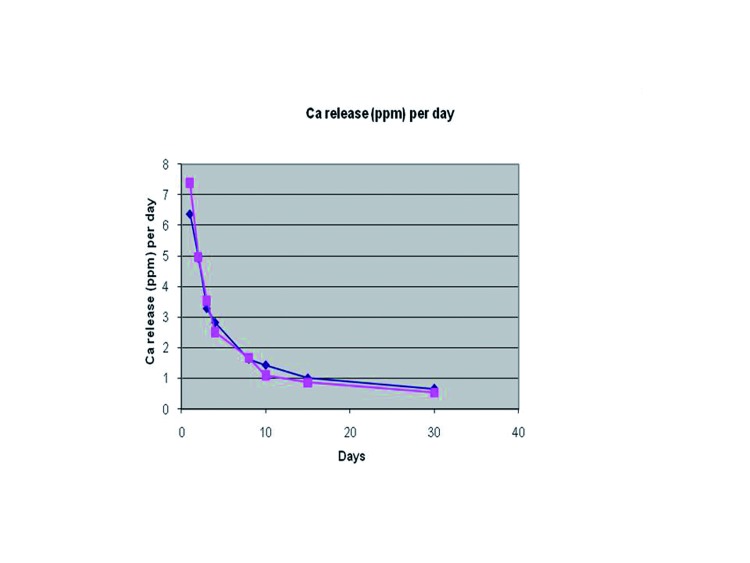
Comparison between PE and C.P.M. of obtained values of Ca2+ release as a function of immersion time.

Both cements released calcium to the water solution, but there were no differences between them (F=0.716; df=1; *p*=0.407). Calcium release significantly changed across time (F=33.770, df=3.392; *p*=8.40•10-15). Release decreased up to day 5 and then slightly recover at 1 month, with a peak on day 8. Differences in calcium release across time were similar for both cements (F=1.265; df=3.392; *p*=0.292) (Fig. 3).

## Discussion

There are many published reports regarding the chemical, physical, and antibacterial properties of MTA (4). Our search showed that MTA is composed of calcium, silica, and bismuth.

The patented material is authorized as Portland cement Type I by the American Standards for Testing Materials (ASTM) with a 4:1 ratio of added oxide bismuth to add radio-opacity to the material. This cement in contact with osseous tissue is supposed to transfer calcium ions to surrounding tissues, a theorically beneficial clinical action. Similarly to MTA, PE cement, as a result of hydration, results in calcium hydroxide surrounding dicalcium and tricalcium silicates. In the initial hours, when the cement powder is mixed with water, the resulting calcium hydroxide dissociates in aqueous calcium and hydroxyl ions. This increases both the pH and concentration of calcium. Camilleri (5) in their study on the hydration mechanism of MTA reported on the ability of processing the material with water and the formation of new elements, comparing them with those of Portland cement. The conclusion was that the microstructure of hydrated MTA is probably more stable than Portland cement. This report suggested that bismuth could affect the hydration mechanism of MTA and therefore precipitation toform calcium hydroxide. A similar methodology to ours was already used by Santos *et al.* (6) to evaluate the release of calcium ions from MTA-Angelus® and a new experimental cement. Both material and time interacted to influence the pH, and also the release of calcium and conductivity. There was also a high correlation between pH, conductivity and calcium regardless of the type of cement, as our data suggest. Besides, the present study adds a measurement after 30 days. Other authors (7) have compared ProRoot® and MTA Angelus® and concluded that differences in pH and calcium ion release do not change substantially with time. However, inmmersion rates in this study is considerably shorter (maximum 3 days). Information on the one-month pH, conductivity and calcium release seem to be relevant for the assessment of the performance of Portland-based cements. To evaluate the radiopacity (8) setting time, pH level, calcium ion release and solubility of white mineral trioxide aggregate (MTA; Angelus, Londrina, Pr, Brazil) with different powder-to-water ratios. Three MTA groups were prepared using 4:1, 3:1 and 2:1 powder-to-water ratios. For the radiopacity analysis, the 10×1mm specimens were arranged on occlusal films with a cylinder of dentine and an aluminium stepwedge. The digitized radiographs were evaluated with Digora 1.51 software to determine the radiographic density. Thirty acrylic teeth with root-end filling material were immersed in ultra pure water for measurement of pH level and calcium ion release (atomic absorption spectrophotometer) at 3, 24, 72 and 168 h. In the solubility test, the root-end fillings of 30 acrylic teeth were scanned twice by a Micro-CT, before and after immersion in ultra pure water for 168h. Digital data were reconstructed, and the volume (mm3) of the samples was obtained using CTan software (CTan v1.11.10.0, SkyScan). The radiopacity was higher (*P*<0.05) when the 4:1 proportion was utilized. The setting time was longer, and the pH level and calcium ion release were higher (*P*<0.05) with a greater volume of water. The group with more water (2:1) had significantly (*P*<0.05) more material volume loss (6,46%) compared with the other groups. The ratio of powder/water significantly interfered with the physical and chemical properties of white MTA Angelus. The influence (9) of additives on several physical and chemical properties of a novel endodontic cement based on calcium aluminate in comparison with mineral trioxide aggregate (MTA) to evaluate. The calcium aluminate cement without additives had a setting time of approximately 60 min, and when combined with Li2CO3 it decreased to 10 min. The material also released Ca2+ ions and alkalinized the medium. The novel cement set more rapidly, had better fluidity, improved handling properties, higher mechanical strength, and reduced porosity with lower pore size compared to Gray-MTA Angelus. The purpose of study (10) was to evaluate the hydrogenionic potential and electrical conductivity of Portland cements and MTA, as well as the amount of arsenic and calcium released from these materials. In Teflon molds, samples of each material were agitated and added to plastic flasks containing distilled water for 3, 24, 72 and 168 h. The electrical conductivity of the cements were not statistically different (*p*>0.05). White non-structural cement Amta-BIO released the largest amount of calcium ions into solution (*p*<0.05), while arsenic release was insignificant in all of the materials (*p*>0.05). The results indicated that the physico-chemical properties of Portland cements and MTA were similar. Further more, all materials produced an alkaline environment and can be considered safe for clinical use because arsenic was not released. The electrical conductivity and the amount of calcium ions released into solution increased over time. The effect of storage pH on solubility (11) of white mineral trioxide aggregate (WMTA), bioaggregate (BA), and nano WMTA cements. Forty-eight moulds randomlyal located in to three groups of pH 4.4 (group A), 7.4 (group B), and 10.4 (group C); and one empty as control in each group. Each group was further divided in to three subgroups according to the material studied; WMTA, BA, and nano WMTA. The specimens in subgroup A were soaked in but yric acid buffered with synthetic tissue fluid (STF) (pH 4.4), while the samples in subgroups B (pH 7.4) and C (pH 10.4) buffered in potassium hydroxide for 24 h and then the loss of cement was determined. Acidic environments can significantly increase the cement loss of all three types of tested materials. However, these cements showed the minimal solubility in alkaline pH values. Nano WMTA showed the lowest cement loss in comparison with WMTA and BA, especially in low pH value. This issue can suggest nano WMTA to be applied in acidic environments such as preapical inflammation.

## Conclusions

Both cements raised the pH of saline solution, although pH decreased with time. Both cements raise the conductivity of saline solution on the first day, which then decreased.

However, pH and conductivity was slightly higher in saline containing PE than in saline with C.P.M.

The calcium release of the PE cement was similar to that of C.P.M. After 30 days the calcium ion release by PE was greater than C.P.M. Both cements still released calcium ions after 30 days of storage in a saline solution.
